# Expert consensus on dental caries management

**DOI:** 10.1038/s41368-022-00167-3

**Published:** 2022-03-31

**Authors:** Lei Cheng, Lu Zhang, Lin Yue, Junqi Ling, Mingwen Fan, Deqin Yang, Zhengwei Huang, Yumei Niu, Jianguo Liu, Jin Zhao, Yanhong Li, Bin Guo, Zhi Chen, Xuedong Zhou

**Affiliations:** 1 State Key Laboratory of Oral Diseases & Department of Operative Dentistry and Endodontics, West China Hospital of Stomatology, National Clinical Research Centre for Oral Diseases, Sichuan University, Chengdu, China; 2 The State Key Laboratory Breeding Base of Basic Science of Stomatology (Hubei-MOST) & Key Laboratory of Oral Biomedicine Ministry of Education, School and Hospital of Stomatology, Wuhan University, Wuhan, China; 3 Department of Cariology and Endodontology, Peking University School and Hospital of Stomatology & National Clinical Research Center for Oral Diseases & National Engineering Research of Oral Biomaterials and Digital Medical Devices & Beijing Key Laboratory of Digital Stomatology, Peking University, Beijing, China; 4 Department of Operative Dentistry and Endodontics, Hospital of Stomatology, Guanghua School of Stomatology, Sun Yat-sen University and Guangdong Provincial Key Laboratory of Stomatology, Sun Yat‑Sen University, Guangzhou, Guangdong China; 5 School of Medicine, Jianghan University, Wuhan, China; 6 College of Stomathology, Chongqing Medical University, Chongqing, China; 7 Department of Endodontics, Shanghai Ninth People’s Hospital, College of Stomatology, Shanghai JiaoTong University School of Medicine, National Clinical Research Center for Oral Diseases, Shanghai Key Laboratory of Stomatology & Shanghai Research Institute of Stomatology, Shanghai Jiao Tong University, Shanghai, China; 8 Department of Endodontics, The First Affiliated Hospital of Harbin Medical University & Department of Endodontics, School of Stomatology, Harbin Medical University, Harbin, China; 9 Key Laboratory of Oral Disease Research, School of Stomatology, Zunyi Medical University, Zunyi, China; 10 Department of Endodontics, First Affiliated Hospital of Xinjiang Medical University, and College of Stomatology of Xinjiang Medical University, Urumqi, China; 11 Affiliated Stomatological Hospital of Kunming Medical University, Kunming, Yunnan China; 12 Department of Stomatology, the First Medical Centre, Chinese PLA General Hospital, Beijing, China

**Keywords:** Dental caries, Caries risk assessment

## Abstract

Dental Caries is a kind of chronic oral disease that greatly threaten human being’s health. Though dentists and researchers struggled for decades to combat this oral disease, the incidence and prevalence of dental caries remain quite high. Therefore, improving the disease management is a key issue for the whole population and life cycle management of dental caries. So clinical difficulty assessment system of caries prevention and management is established based on dental caries diagnosis and classification. Dentists should perform oral examination and establish dental records at each visit. When treatment plan is made on the base of caries risk assessment and carious lesion activity, we need to work out patient‑centered and personalized treatment planning to regain oral microecological balance, to control caries progression and to restore the structure and function of the carious teeth. And the follow-up visits are made based on personalized caries management. This expert consensus mainly discusses caries risk assessment, caries treatment difficulty assessment and dental caries treatment plan, which are the most important parts of caries management in the whole life cycle.

## Introduction

Dental caries is a common chronic infectious disease that occurs in the dental hard tissues. Dental caries and its complications can exacerbate or induce systemic diseases, which seriously reduce the quality of human life and cause a great economic burden. According to current investigations, there are still great challenges in dental caries prevention and treatment. Firstly, the prevalence of dental caries is very high. The results of the global burden of disease study released by Lancet in 2017 showed that among 328 diseases, the prevalence of permanent dental caries ranked first, and the incidence ranked second.^1^ There are around 2.44 billion population worldwide suffering from permanent tooth decay. Besides, The 4th National Oral Health Survey in the Mainland of China shows that the prevalence of deciduous tooth caries in 5-year-old children is 71.9%, which is 5.9% higher than that of 10 years ago, and the prevalence of permanent tooth caries in 12-year-old children is 38.5%, which is 9.6% higher than that of 10 years ago.^2^ Secondly, the ratio of treated caries is quite low. In 2017, 7.8% of the global population had untreated deciduous tooth caries, while those with untreated permanent tooth caries accounted for 29.4% of the global population.^3^ Thirdly, the failure rate of dental restorations is rather high. In the follow-up cases, the total failure rate of 1821 restorations was 24.1%, and 10-year survival rates for Class III and Class IV restorations were reported to be 95 and 90%, respectively.^4^ Therefore, we still need to make effective strategies to combat dental caries in clinical practice.

## Dental caries management

**Fig. 1:**
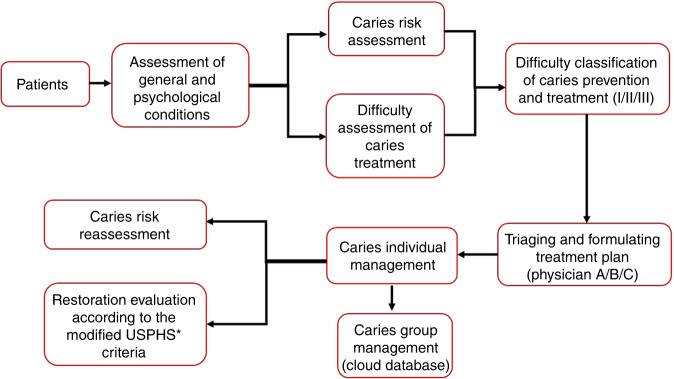
Process of difficulty assessment of dental caries prevention and treatment. ^*^USPHS: United States Public Health Service Commissioned Corps

In the aspect of individualized management of patients with dental caries, traditional prosthetic treatment lacks comprehensive management of prevention and treatment based on risk assessment and difficulty assessment for it is mainly based on the “drill and fill” model. Carrying out caries risk assessment (CRA) for patients, analyzing and controlling risk factors for caries occurrence, and formulating personalized caries treatment and management plans on account of CRA have become the new trend of modern dental caries management.^5^ At present, there are several classification and management standards of dental caries being widely applied in the world. The International Caries Detection and Assessment System (ICDAS) was established in 2002,^6^ and in 2009, caries activity tests were added to develop the modified clinical caries classification standard—ICDAS‑II.^7^ Based on the ICDAS, the International Caries Detection and Evaluation System Collaboration Committee proposed the International Caries Classification and Management System, ICCMS. Recently, we proposed the dental caries management should be carried out in the whole life cycle for the first time. The physiological features of patients at different ages should be considered and personalized management plan of dental caries should be made according to different risk factors and risk levels^8^ (Fig. 1).

### Caries risk assessment

The caries risk assessment is one of the most important elements of dental caries management. It has an impact on the difficulty assessment of caries prevention as well as making treatment plan before dental caries treatment; and the risk re-assessment after treatment is related to the curative effect and prognosis, which is also significantly important in caries management. And there are several dental caries risk assessment systems worldwide, including American Dental Association (ADA) caries risk assessment, Caries risk assessment tool (CAT), Caries management by risk assessment (CAMBRA) and Cariogram. And the caries risk assessment forms of ADA, CAT and CAMBRA are presented in Tables S6–S10.

#### American Dental Association (ADA) caries risk assessment^9^

This risk assessment system was proposed by the ADA in 2004 and is divided into two forms. One form is for patients ages 0–6 years of age and the other is for patients over 6 years of age. It mainly includes three aspects: contributing conditions, general health conditions, and clinical conditions. Contributing factors refer to external factors that could influence the occurrence and development of dental caries; general health conditions refer to the physical conditions of patients; and clinical conditions refer to intraoral conditions that directly related to dental caries. The system divides caries risk into high, moderate, and low grades, and is mainly used to assist dentists in assessing patient’s caries risk in clinical practice.

#### Caries-risk assessment tool (CAT)^10^

CAT is developed by the American Academy of Pediatric Dentistry and is divided into 2 forms: (1) for children aged 0–5 (dental practitioners, physicians, and other non-dental health care providers); (2) for children ≥ 6 years old, adolescents and adults (used by dental providers). CAT’s evaluation indicators cover risk factors (social/biological), protective factors and clinical findings, and it is mostly used for CRA in infants, children and adolescents.

#### Caries management by risk assessment (CAMBRA)^11^

CAMBRA was first proposed by the California Dental Association in 2002, and was modified afterwards to form the existing format. It consists of two tables: 0–6 years old and over 6 years old. And its assessment criteria include pathological indicators, risk factors, protective factors and bioprotective factors.

#### Cariogram^12^

Cariogram is a computer-programmed CRA system developed by Swedish scholars and is composed of 9 caries risk factors. The system can perform weighted analysis on the input data and apply a pie chart to predict the patient’s the overall caries risk; meanwhile, it can demonstrate the respective influences of different risk factors, predict the individual possibility of developing caries in the future, and propose targeted methods to prevent new caries.

The CRA systems mentioned above are applicable to population aged 0–6 years old and ≥6 years old (Table S1, S2). Among them, CAMBRA system covers the largest number (#25) of factors related to caries for adults, followed by ADA (#19) and Cariogram (#14).^13–17^ CAMBRA system also suggests the largest number (#20) of factors associated with dental caries for children, then followed by ADA (#14) and CAT (#13), and Cariogram (#9). Studies had revealed that Cariogram had a limited extent in predicting dental caries in preschool children, but more useful in identifying caries risk for the elderly.^18^ Gao et al.^19^ reported a high sensitivity and low specificity of CAT and CAMBRA for 3-year-olds children, but could not effectively predict the occurrence of new caries. Such low specificity may overestimate the level of children’s caries risk, which might lead to overtreatment and raise the cost of prevention. The ADA assessment system is also most commonly used in children, but there is still a lack of relevant research to confirm its caries prediction ability in all age groups.

Accurate and valid caries risk assessment can provide support for effective caries management, so as to implement targeted and progressive measures regarding caries prevention and treatment.^20^ However, the accuracy of caries risk assessment for children is still low, and there is still a lack of risk assessment guideline for low-income population. There are deficiencies among these assessment systems and certain limitations in the range of application.^21,22^ Therefore, appropriate assessment methods should be selected or adjusted according to patients’ age, region and other specific conditions. Currently, there is still a lack of multifactorial caries risk assessment system in China, and more research are needed to identify and evaluate whether the existing caries risk assessment system is suitable for Chinese populations.

### Classification and activity of caries

Caries is a dynamic development process. If the demineralization process of caries is in progress, which is accompanied by the rapid loss of calcium and phosphorus ions, it is called active caries. Otherwise, if the demineralization process stops, which means the chemical reaction has ended, it is called arrested caries. Besides, carious lesion activity could be classified according to surface characteristics. The enamel of active caries typically appears whitish or yellowish change with loss of luster, and the texture feels soft on probing. It often locates in the pit and fissure, the gingival margin and contact points of the proximal surface, which is generally covered with plaque. Dentine caries usually appears brownish. The surface of the cavity feels soft on probing, and it is cheese-like and fragile. For arrested caries, the enamel is whitish or brownish. The surface is smooth and feels hard on probing, and no obvious plaque on the surface. And the dentin appears typically dark brown. The surface of the cavity feels hard and leathery on probing.

Because carious management is related to the caries diagnostic criteria, we should recognize the importance of carious lesion activity. Nyvad^23^ previously proposed a detailed caries diagnostic criterion in 1999, distinguishing the active and inactive caries based on a combination of visual and tactile criteria (Table S3). This assessment was carried out at three levels of increasing severity which scored from 0 and 9.

In 2002, an international team of caries researchers harmonized global evidence around caries detection and assessment to create a standard system named the International Caries Detection and Assessment System (ICDAS).^6,24^ This system encodes caries depending on the minor variations in visual signs of the lesion severity and the radiographic information for the depth of caries demineralization (Table S4).^6,25^ which classifies caries effectively and is helpful for the early diagnosis of caries, but does not involve carious lesion activity.

ICDAS-Caries Lesion Activity Assessment (also called in the literature ICDAS-CAA for ICDAS-Clinical Characteristic Assessment) was proposed in 2009, which is based on combinations of visual (appearance and plaque stagnation) and tactile criteria (Table S5).^26,27^

ICDAS system has been widely used and constantly improved in European and American countries, which subsequently developed into International Caries Classification and Management System (ICCMS). ICCMS synthesizes the radiographic and clinical assessment to categorize the lesions with the ICDAS merged codes, which divides coronal caries into sound surfaces (ICDAS^TM^ code 0), initial stage caries (ICDAS^TM^ codes 1 and 2), moderate stage caries (ICDAS^TM^ codes 3 and 4) and extensive stage caries (ICDAS^TM^ codes 5 and 6).^28^ Combined with the lesion activity assessment, the ICCMS diagnostic classification of caries includes no lesion, initial inactive lesion, initial active lesion, moderate active lesion, moderate inactive lesion, extensive active lesion and extensive inactive lesion.

For the advantages of ICDAS-CAA: (1) easy to use; (2) no special equipment required; (3) the changes of lesions can be followed up; (4) low cost. However, this system also has some limitations: (1) there is a certain degree of subjectivity; (2) the tooth surface must be cleaned before use; (3) inspectors must be trained and calibrated.

Both the Nyvad criteria and ICDAS-CAA show relatively good intrinsic validity. For Nyvad criteria, which shows sensitivity at 0.71, the specificity is between 0.73 and 0.75, the intra-examiner reproducibility (Kappa value) is between 0.68 and 0.80, and the inter-examiner reproductivity is between 0.74 and 0.90. ICDAS-CAA, which is part of the ICCM^TM^, shows sensitivity at 0.87 in enamel and 0.60 in dentin, specificity at 0.50 in enamel and 0.95 in dentin, the intra- and inter-examiner reproducibilities (Kappa values) are between 0.11 and 0.96, and between 0.20 and 0.95, respectively. However, in the daily practice of general practitioners, Nyvad criteria and ICDAS-CAA are rarely used, further in vivo investigations with Nyvad criteria and ICDAS-CAA as gold standards are needed.^27^

The clinical significance of evaluating carious lesion activity is that identifying active lesions during clinical examination could directly help carious management and follow-up monitoring, especially for the caries with severe progression. For non-cavitated active caries, oral hygiene guidance could be carried out for patients, non-surgical interventions (oral hygiene instruction, topical application of fluoride) could be carried out according to individual conditions. Besides, no surgical intervention is required for arrested caries, except basic preventive measures (brushing with fluoride toothpaste). And many clinical studies have confirmed that non-surgical treatment can effectively control caries.^29^ Once active caries has affected dentin, it must be treated with restorations. For active caries, it is necessary to formulate a combined treatment plan. The carious management plan at the individual level could reduce the risk of caries and prevent future caries. Moreover, it is also necessary to develop a carious treatment plan for the tooth level, including non-surgical treatment and surgical restorative treatment to manage the existing carious lesion activity.

### Dental caries treatment plan

According to the modern etiology, dental caries results from complex interactions over time between host factors, oral microbe, fermentable carbohydrate. The prognosis of the disease is closely related to the general condition and oral factors. Although there is a relatively well-developed caries management system, the difficulty assessments of dental caries treatment are still needed before making treatment plan. Then caries management plan is conducted to control caries risk factors and manage individual lesions.

#### Difficulty assessment in caries treatment

Based on the difficulty factors of dental caries treatment, we proposed the difficulty assessment of caries prevention and treatment to guide the clinical diagnosis, treatment and referral, provide objective preoperative prediction of treatment outcomes, facilitate communication between dentists and patients and improve the quality of caries treatment and long-term therapeutic effect. The factors affecting the difficulty of caries treatment mainly include systemic and oral factors, individual susceptibility to caries, technical sensitivity, past dental filling experience and auxiliary factors.^30^ The caries factors include the involved carious tooth surface and the depth of lesion, which directly affect the difficulty of caries treatment and treatment decisions; secondly, with the development of materials and methods, the technical sensitivity of the treatment of caries has increased. As for technical factors, the main techniques commonly used in clinical practice such as non-surgical treatment, direct and indirect restorative treatment are scored; the treatment for secondary caries and old restorations is one of the difficult points affecting the treatment of caries, so the history of tooth restoration and failure of dental restoration are also the main contents of the difficulty evaluation; other factors, such as mouth opening, pharyngeal reflex, saliva secretion and dental phobia, can directly or indirectly affect the difficulty of caries treatment, so they were used as an additional factor to assess the difficulty of caries treatment.

According to the difficulty of treatment, each factor is divided into Level 1-3 and the comprehensive assesment is divided into I, II and III grades. Dentists are also classified into A, B and C levels according to their technical proficiency. Combined the difficulty assessment grades and the referral advice, caries risk difficulty classification can be divided into low risk, medium risk, high risk and extremely high risk levels (Table1). The specific grades are as follows:

**Table 1:** Difficulty assessment of dental caries treatment

Difficulty classification	Level 1	Level 2	Level 3
Involved tooth surface and site	Class I and V	Class II, III, IV and VI Root caries (involving labial/buccal surface)	Cavity on the 1/3 gingival side of the distal surface of posterior teeth Attrition Cusp defect Severely defected crown Root caries (involving more than 2 surfaces) Rampant Caries
Depth of caries lession	Superficial caries and intermediate caries	Deep caries	Deep caries of immature permanent teeth
Technique types	Direct restoration of posterior teeth: composite resin restoration and amalgam restoration Minimally invasive techniques: ART, preventive resin restoration (PRR), glass ionomer transition repair, enamel molding, and micro-polishing	Composite resin restoration of anterior teeth	Cosmetic restoration of anterior teeth: non-invasive esthetic restoration, minimally invasive layered restoration, minimally invasive CAD/CAM ceramic veneer restoration Inlay restoration of posterior teeth: composite resin inlay, CAD/CAM ceramic inlay restoration
History of restoration or filling failure	A history of restoration, but caries not affecting the old restoration	Caries involving the old restoration or the first fracture of the old restoration	Old restoration falling off 2 or more times
Mouth opening	3 fingers wide	2 fingers wide	Less than 2 fingers wide
Pharyngeal reflex^a^	No	Yes	Strong
Salivary secretion^b^	Normal	Many	Excessive
Dental phobia	No	Yes	
Caries risk assessment^c^	Low and medium risk population	High risk population	Extremely high risk population

^a^Pharyngeal reflex: “no”, treatment of caries can be completed without special assistance; “yes”, patient’s pharyngeal reflex is obvious, but the caries treatment can be successfully completed with special assistance (such as rubber barrier); “strong”, it is also difficult to complete caries treatment with special assistance.

^b^Salivary secretion: “normal”, the treatment can be successfully completed under the gauze ball isolation; “many”, it is difficult for the yarn ball to block moisture and requires four-handed operation; “excessive”, rubber barriers must be placed.

^c^Classification of caries risk assessment: According to CAMBRA caries risk assessment model, patients will be divided into low risk, medium risk, high risk and extremely high risk population.

Grade I: Preoperative evaluation of the cases is not difficult, and all the difficulty factors are in line with level 1 of difficulty assessment system of caries prevention and management. Doctors with low experience are competent for the diagnosis and treatment of these cases. This group of patients should be referred to grade A doctors (general practitioners).

Grade II: The preoperative evaluation showed that the cases are difficult, and one difficulty factor is in accordance with level 2 of difficulty assessment system of caries prevention and management. Even experienced dentists may face challenges in diagnosing and treating these cases. Such patients are supposed to refer to grade B doctors (cariology specialists).

Grade III: Preoperative evaluation of the cases is difficult, with at least 2 difficulty factors matching level 2 or 1 difficulty factor matching level 3 of difficulty assessment system of caries prevention and management. Experienced doctors also face challenges in achieving the desired outcome. Those patients should be referred to grade C doctors (clinical experts in cariology).

#### Caries lesion management

Caries treatment planning is a serialized process that aims to eliminate or control pathogenic factors, restore existing lesion, and produce a functional and sustainable environment. The essential steps include clinical examination, definite diagnosis, risk assessment, devising optimal treatment plan, delivering alternative plan and patient-participated decision making.^31^

Decision-making process and personalized caries care plan are based on accurate diagnosis. At the caries level, the diagnostic process should include caries detection, assessment of caries severity (e.g., penetration depth of the lesions, with or without cavities) and caries activity (i.e., active or inactive). Note that during the diagnosis phase caries risk assessment is also conducted.^32^

Caries management includes two aspects—controlling caries risk factors and managing individual lesions. Caries management is patient-centered and based on caries risk assessment, which takes comprehensive measures such as health promotion, prevention or treatment to affect various factors of caries occurrence and development, in order to regain oral microecological balance, control caries progression and restore the structure and function of the teeth.^33^ Overall, caries management refers to the use of interventions to stop the progression of existing lesions and non-self-cleaning active caries, aims at controlling the development of caries at the tooth level.^34^

According to the different tissues (i.e., enamel or dentin) and surfaces (e.g., occlusal, proximal, and root) in which the caries is located, different interventions are required.^35^ Besides, lesions activity also influences the need for interventions. The transformation from active caries to inactive can happen through exposure to saliva, self-cleaning, and so on, or can be aided with products and/or interventions.^36^

##### Non-cavitated caries lesions

Guided by the caries risk assessment results, caries category (ICDAS 1~2) and caries activity, individual caries treatment plan can be formulated and corresponding caries management measures can be taken.^5^ Non-cavitated inactive caries lesions do not need operative care, while the treatment methods of active caries are different from caries locations.^36^

Dental sealant is considered to be the most cost-effective treatment for the prevention of pit and fissure caries.^35,37^ According to the 2018 American Dental Association’s (ADA) systematic review and subsequent evidence-based clinical practice guideline, Pit and fissure sealant has been recommended as the treatment method for non-cavitated pit and fissure caries.^38,39^ Sealant can be used alone or in combination with 5% NaF varnish (application every 3–6 months), and the combined approach has been confirmed as the effective intervention in arresting or reversing lesions.^39^ Additionally, 5% NaF varnish (application every 3–6 months), 1.23% acidulated phosphate fluoride (APF) gel (application every 3–6 months) or 0.2% NaF mouth rinse (once per week) can be considered as a suboptimal treatment strategy for non-cavitated pit and fissure caries.

Resin infiltration is recommended as the non-invasive management of non-cavitated approximal caries lesions.^40^ Both applying resin infiltration alone and resin infiltration plus 5%NaF varnish every 3–6 months could effectively prevent non-cavitated caries lesions process on approximal surfaces.^39^ In addition, dental sealant can also be applied to non-cavitated approximal caries, but the clinical operation is difficult due to the need of special instruments and high technical sensitivity.

For non-cavitated caries lesions on facial or lingual surfaces, 1.23% APF gel (application every 3–6 months) or 5% NaF varnish (application every 3–6 months) is recommended.^35^

In order to arrest the caries progress and promote remineralization, various forms of calcium-containing products came into being. However, due to the clinical efficacy limitations, calcium-containing agents can not be applied as a substitute for fluoride in the treatment for non-cavitated caries.^41^

##### Cavitated caries lesions

The caries risk factors management plan is tailored at the individual caries risk assessment result, and the caries lesions management strategy depends on the lesions severity and caries activity status. Compared with non-cavitated caries, the management of cavitated caries lesions increases the restorative treatment plan. Some cavitated caries lesions that do not invade dental pulp can take the nonrestorative treatment with silver diamine fluoride (SDF) temporarily or permanently, when the main purpose is to arrest the progression of caries regardless of functional and esthetic effects. Studies indicated that applying SDF every 6–12 months could effectively arrest the cavitated caries process.^38,39^ However, nonrestorative treatment measures have limitations, and cavitated caries lesions are generally noncleansable and active. Therefore, restorative treatment is the main intervention strategy for cavitated caries, which is aimed to control the biofilm in specific locations, seal the crown with adhesive materials, protect the dentin-pulp complex, terminate the activity of lesions as well as restore the function, shape and esthetic. For moderate stage caries (ICDAS 3~4), minimally invasive restorative treatments are carried out on the basis of controlling plaque as well as reducing caries risk and lesions activity. Selective removal to firm dentine is the treatment of choice for moderate stage caries in order to maintain the restoration longevity, while selective removal to soft dentine is recommended in deeper cavitated lesions to give priority to preserve pulpal health.^42^

##### Deep caries

Deep lesions are defined as those radiographically involving the inner pulpal third or quarter of dentine or with clinically assessed risk of pulpal exposure,^43^ which are similar to extensive stages caries (ICDAS 5~6). The treatment principles of deep caries include arresting the caries process, promoting pulp defensive response and giving priority to the preservation of pulp. Carious removal in deep caries should follow the principle of minimally invasive and gradual, which requests to use the hardness of the remaining dentine as the criterion in assessing the end point of carious tissue removal for cavity. Application of rubber dam were recommended to maintain an aseptic environment. Cavity disinfection is not a necessary means as there is insufficient evidence to support it. What’s more, magnification is advantageous to determine the end point of carious tissue removal and pulp exposure or not. Selective removal to soft dentine is recommended in deep caries lesions, in order to retain non-demineralized or remineralizable tissue, and maintain the vitality of dental pulp.^44^ Soft dentine is defined as that it will deform when a hard instrument is pressed on and can be easily scooped up.^43^

Stepwise technique (SW) can be used on deep carious lesions. The first step in the SW is the procedure by which carious dentine is removed from the peripheral walls to hard dentine (a scratchy sound or ‘cri dentinaire’ can be heard in hard dentine when a straight probe is taken across), followed by excavation that soft carious tissue was left in the pulpal aspect of the cavity.^45^ Calcium hydroxide cement then is applied over the pulpal wall with high strength glass-ionomer cement (GIC) sealing the cavity temporarily. At the meantime, it is necessary to strengthen caries management measures and observe the symptoms. Dental visit after 6–12 months if the symptoms are improved or without obvious symptoms; In case of spontaneous pain, make an appointment for return at any time. The second treatment is performed after an interval of 6–12 months.^43^ If the symptoms disappear, remove all the GIC, excavate until only leathery/firm dentine (this kind of dentine is physically resistant to hand excavation, and some pressure needs to be exerted through an instrument to lift it) remains over the pulp.^42^ Then using selective etch adhesion technology and composite resin for restoration. It is necessary to implement caries management measures conventionally and maintain recalls at risk-based intervals.

SW as an early caries removal technique, calcium hydroxide is still the most commonly used indirect pulp capping materials in it.^46^ New materials such as hydraulic calcium silicates (hCSCs) have been developed, in particular various forms of the mineral trioxide aggregate (MTA), and another recent available type Biodentine and iRoot BP. Although recent reviews^47,48^ provide the evidence for a more superior outcome for the biological properties and material advantages of hCSCs than calcium hydroxide, there remains insufficient evidence comparing and testing these indirect pulp capping materials in order to make definitive conclusions on the best material to use.

There are some evidences have suggested that the second removal step may be omitted, as it increases risk of pulpal exposure. In addition, it increases the cost, treatment time and uncomfortable feelings.^49–51^ A randomized clinical trial has showed that the success rates for SW were 93% and 69% after 1 and 3 years follow-up, respectively, while partial caries removal group were 98% and 91%. The comparison between two groups showed statistically significant differences, which may be explained by the high number of uncompleted SW treatments.^52^

##### Restorative materials

Direct restorative materials mainly contain GICs and composite resin. Due to the emission of mercury in the production and use of silver amalgam, the United Nations has formulated and issued a convention, requiring measures to phase down the use of silver amalgam.^53^ The materials selection varies according to the remaining coronal tooth tissue, the size of the restoration, occlusal forces, caries risk, and esthetics.^54^

GIC has good biocompatibility, binds chemically to dental hard tissues, releases fluoride, may protect against secondary caries.^55,56^ High viscosity glass-ionomer cements (HV-GICs) have been promoted in recent years, which have similar bond strength to both normal and caries-affected dentin. Unlike HV-GICs, resin adhesives have a significantly lower bond strength to caries-affected dentin than sound dentin.^57^ Therefore, HV-GIC is recommended as temporary restoration after selective removal of caries to soft or leathery dentin.^58^ Patients with xerostomia after radiation treatment will be classified as high caries risk with high likelihood of caries incidence. Multiple studies have shown that restorative treatment with HV-GIC in these patients has a good survival rate, and can protect against secondary caries even in the case of low fluoride compliance or the restoration falls off.^59–62^ For the patients in high caries risk level, it is recommended to restore with GIC first, and then composite resin can be applied after caries risk factors are controlled. What’s more, recent system review suggested no difference in the failure rates between HV-GIC and hybrid resin composite restorations.^63^

The prevention of root caries is more important than treatment, in view of the high morbidity of root caries among older population. Due to the rapid progress of root caries, the carious tissue must be removed as soon as possible to protect the pulp once caries occurs. It suggests clinicians prioritize the use of fluoride-releasing GIC for root caries treatment. If the root caries located at the anterior region where the esthetic factors should be considered, it can be restored by composite resin after the cavity lining with the GIC.

For the restoration of anterior region caries, attention should be paid to recover the beauty by using esthetic restoration measures. Composite resin is often used in the restoration of anterior region defects due to the minimally invasive, repairable and esthetic features. When the caries is involved in the incisal angle, the lingual wall can be restored with paste-like resin or flowable resin in conjunction with the guide plate. Dentin shade resin is used to restore the dentin above the lingual wall. The incisal edge is restored with transparent shade resin, and the enamel surface with enamel shade resin.

Composite resin is commonly applied for posterior region restoration, and GIC can also be used under special circumstances. The selection of composite resin materials can be based on the location and depth of the cavity. Flowable composite resin can be applied to superficial pit and fissure caries,^64^ while for caries cavities whose depth of penetration is greater than 2 mm, high-viscosity or viscosity variable bulk-fill resin can be applied for one step filling. On the other hand, the low-viscosity bulk-fill resin has greater fluidity and lower mechanical properties than that of high-viscosity type, which needs to be covered with a layer of traditional composite resin after filling. The application of low-viscosity bulk-fill resin needs at least two steps filling to complete clinical operation, which increases the operation steps.

**Fig. 2:**
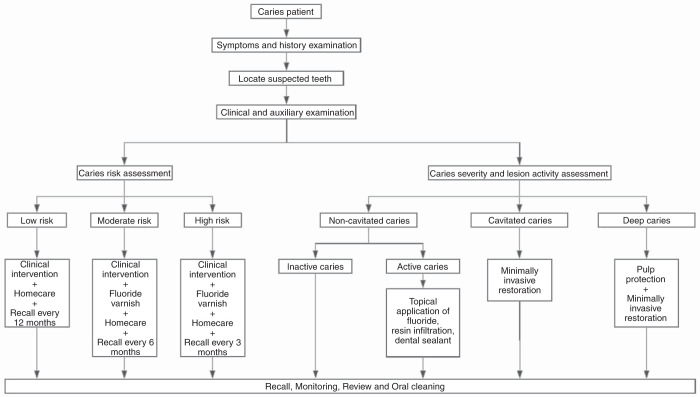
Clinical decision model for caries management^57^

Overall, clinicians should synthesize the assessments of caries risk, caries severity and lesion activity to make personalized caries management plan (Fig. 2),^65^ so as to provide targeted personalized diagnosis and treatment for patients.

### Assessment and management of caries after treatment

After caries treatment, further management should be strengthened to improve the prognosis of caries treatment and reduce the risk of new caries. Caries risk assessment is throughout the whole management process before and after caries treatment. For example, CAMBRA requires dentists to put forward professional oral health care advice and implement corresponding management measures according to the assessment of patients’ caries risk (low, moderate, high and extreme) (Table 2).^66^ Regularly follow-ups (1, 6, 12 months) are required after the restorative treatment. Dentists could evaluate the treatment and the new caries risk by using the modified U.S. Public Health Service standard and update corresponding management measures combined with caries risk assessment system.^67^ Thus a cycle of management is formed.

**Table 2:** Caries Management by Risk Assessment according to CAMBRA^66^

Risk Level	Antibacterials	Saliva Test (Saliva flow & bacterial Culture)	Fluoride	Frequency of radiographs	Frequency of caries recall Exams	Xylitol & soda water	Sealants
Low risk	Not required	May be done as a base line reference for new patients	OTC fluoride-containing toothpaste twice daily	Bitewing radiographs every 18–24 months	Every 6–12 months to reevaluate caries risk	Not required	Not required
Moderate risk	Not required	May be done as a base line reference for new patients or if there is suspicion of high bacterial challenge	OTC fluoride-containing toothpaste twice daily plus: 0.05% NaF rinse daily	Bitewing radiographs every 12–18 months	Every 4–6 months to reevaluate caries risk	Two tabs of gum or two candies four times daily	Required
High risk	Chlorhexidine gluconate 0.12% 10 ml rinse for one minute daily for one week each month	Saliva flow test and bacterial culture initially and at every caries recall appointment	1.1% NaF toothpaste twice daily instead of regular fluoride toothpaste. NaF varnish clinically	Bitewing radiographs every 6–12 months or until no cavitated lesions are evident	Every 3–4 months to reevaluate caries risk and apply fluoride varnish	Two tabs of gum or two candies four times daily	Required
Extreme risk	Chlorhexidine 0.12% (preferably CHX in water base rinse) 10 ml rinse for one minute daily for one week each month	Saliva flow test and bacterial culture initially and at every caries recall appointment	1.1% NaF toothpaste twice daily instead of regular fluoride toothpaste. NaF varnish clinically; household fluoride gel tray 5 min daily	Bitewing radiographs every 6 months or until no cavitated lesions are evident	Every 3 months to reevaluate caries risk and apply fluoride varnish	Two tabs of gum or two candies four times daily. Soda rinses four to six times daily	Required

## Conclusion and expectation

It is of great significance to carry out entire-population and full-life-cycle caries management to maintain oral and systemic health and protect natural teeth.^68,69^ With the development of new diagnostic and therapeutic techniques, such as ultrasonic and optical diagnosis, 3D printing and digital navigation, difficulty assessment system of caries prevention and management will be further improved. Based on the research progress of etiology and pathogenesis of caries, core microbiome is considered as the main factor of the occurrence and development of caries and the key to adjust the unbalanced micro ecological targets. It is expected to become the main microbiological index of caries risk assessment.^70–73^ With the deep research of the microbial community and the application of machine learning, the caries prediction can be carried out by the big data of microbial community, which will further enrich the difficulty assessment system of caries prevention and management. Because of the high prevalence of caries in our country, it is essential to effectively integrate family doctors, community doctors, oral general practitioners and cariology specialists, and combine with community management and personalized treatment. Caries risk assessment and difficulty assessment system of caries prevention and management are important basis for hierarchical diagnosis and treatment. Through the promotion of full-life-cycle caries management, medical resources can be used more efficiently to achieve effective caries prevention and treatment.

## Supplementary information

Supplementary Materials for Expert Consensus on Dental Caries Management
