# Supplementary material for: Expert consensus on dental caries management
Source: Int J Oral Sci. 2022 Mar 31;14:17. doi: 10.1038/s41368-022-00167-3 (PMC8971510; doi:10.1038/s41368-022-00167-3)
Supplement: Supplementary file 1 — Supplementary Materials for Expert Consensus on Dental Caries Management [file 41368_2022_167_MOESM1_ESM.docx]

**Expert Consensus on Dental Caries Management**

**Lei Cheng^1#^, Lu Zhang^2#^, Lin Yue^3^, Junqi Ling^4^, Mingwen Fan^5^, Deqin Yang^6^, Zhengwei Huang^7^, Yumei Niu^8^, Jianguo Liu^9^, Jin Zhao^10^, Yanhong Li^11^, Bin Guo^12^, Zhi Chen^2^*, Xuedong Zhou^1^***

^1^ State Key Laboratory of Oral Diseases & Department of Operative Dentistry and Endodontics, West China Hospital of Stomatology, National Clinical Research Centre for Oral Diseases, Sichuan University, Chengdu, China.

^2^ The State Key Laboratory Breeding Base of Basic Science of Stomatology (Hubei-MOST) & Key Laboratory of Oral Biomedicine Ministry of Education, School and Hospital of Stomatology, Wuhan University, Wuhan, China.

^3^ Department of Cariology, Endodontology and Operative Dentistry, School and Hospital of Stomatology, Peking University, Beijing, China

^4^ Department of Endodontics, Guanghua School of Stomatology, Guangdong Provincial Key Laboratory of Stomatology, Sun Yat‑Sen University, Guangzhou, Guangdong, China.

^5^ School of Medicine, Jianghan University, Wuhan, China

^6^ Chongqing Medical University, College of Stomathology, Chongqing, China.

^7^ Ninth People’s Hospital, School of Medicine, Shanghai Jiao Tong University, Department of Endodontics and Operative Dentistry, Shanghai Key Laboratory of Stomatology, Shanghai, China.

^8^ Department of Endodontics, The First Affiliated Hospital of Harbin Medical University & Department of Endodontics, School of Stomatology, Harbin Medical University, Harbin, China.

^9^ School of Stomatology, Zunyi Medical University & Special Key Laboratory of Oral Diseases Research, Higher Education Institution, Zunyi, China.

^10^ Department of Endodontics, First Affiliated Hospital of Xinjiang Medical University (Affiliated Stomatological Hospital), Urumqi, China.

^11^ Yunnan Provincial Key Laboratory of Stomatology, The Affiliated Stomatology Hospital of Kunming Medical University, Kunming, Yunnan, China.

^12^ Institute of Stomatology of Chinese PLA General Hospital, 28 Fuxing Road,Haidian District, Beijing, People's Republic of China.

* The corresponding author.

# These authors contributed equally to this work.

Table S1. Assessment factors and indicators of caries risk assessment systems for population age 0-6

| Assessment System | Factors |
| --- | --- |
| ADA Caries Risk Assessment System (2011, age 0-6)[13] | Contributing conditions: fluoride exposure, sugary foods or drinks, caries experience of family members, etc.; General health conditions (developmental, physical, medical or mental disabilities that prevent or limit performance of adequate oral health care by themselves or caregivers); Clinical conditions: dental caries condition, visible plaque, orthodontic appliances present, salivary flow, etc. |
| CAMBRA (2007, age 0-5)[14] | Pathological indicators: caries experience of family members, parental socioeconomic status, children's physical development problems, etc.; Risk factors: frequency of snack intake, sleep habits (with or without a bottle to sleep), use of drugs that reduce saliva flow, etc.; Protective factors: abiotic protective factors include the absence of caries of the mother or caregiver in the last 3 years, regular oral health care for children. Bioprotective factors include the use of fluorine and sugar substitutes, etc. |
| CAT (2019)[15] | **for 0-5 years old:**  Risk factors, social/biological: mother/primary caregiver has active dental caries, parent/caregiver has life-time of poverty or low health literacy, child has frequent exposure (>3 times/day) between-meal sugar-containing snacks or beverages per day, child use bottle or non-spill cup containing sugar between meals and/or at bedtime, child is a recent immigrant, child has special health care needs; Protective factors: child receives optimally-fluoridated drinking water or fluoride supplements, child has teeth brushed daily with fluoridated toothpaste, child receives topical fluoride from health professional, child has dental home/regular dental care; Clinical findings: child has non-cavitated (incipient/white spot) caries or enamel defects, child has visible cavities or fillings or missing teeth due to caries, child has visible plaque on teeth. |

ADA (American Dental Association); CAMBRA (Caries Management by Risk Assessment) is a caries risk assessment and management system proposed by California Dental Association, which was then modified to the existing model; CAT is a caries-risk assessment and management protocols proposed by American Academy of Pediatric Dentistry.

Table S2. Assessment factors and indicators of caries risk assessment systems for population age >6

| Assessment System | Factors |
| --- | --- |
| Cariogram (2005)[12] | Past carious experience, related diseases, diet (contents), diet (frequency), plaque amount, levels of m*utans Streptococci*, fluoride programme, saliva secretion, saliva buffer capacity. |
| CAMBRA (2007)[16] | Pathological indicators: visible cavities or radiographic penetration of the dentin, radiographic approximal enamel lesions (not in dentin), white spots on smooth surfaces, restorations last 3 years, etc.; Risk factors: detection level of *Streptococci* and *Lactobacilli*, visible heavy plaque on teeth, frequent snack, deep pits and fissures, drug use, inadequate saliva flow, etc.; Protective factors: use of fluorine and sugar substitutes, chlorhexidine prescribed, calcium and phosphate paste, etc. |
| CAT (2019)[15] | **for ≥6 years old (professional use):**  Risk factors, social/biological: patient has life-time of poverty or low health literacy, patient has frequent exposure (>3 times/day) between-meal sugar containing snacks or beverages per day, child is a recent immigrant, patient has special health care needs; Protective factors: patient receives optimally-fluoridated drinking water, patient brushes teeth daily with fluoridated toothpaste, patient receives topical fluoride from health professional, patient has dental home/regular dental care; Clinical findings: patient has ≥1 interproximal lesions, patient has active non-cavitated (white spot) caries lesions or enamel defects, patient has low salivary flow, patient wears an intraoral appliance. |
| ADA (2011)[17] | Contributing conditions: fluoride exposure, sugary foods or drinks, caries experience of family members, etc.; General health conditions: chemo/radiation therapy, eating disorders, medications that reduce salivary flow, drug/alcohol abuse; Clinical conditions: dental caries condition, visible plaque, tooth morphology, exposed root surfaces, orthodontic appliances present, salivary flow, etc. |

Cariogram is a computerized caries risk assessment system developed by Swedish researchers; CAMBRA (Caries Management by Risk Assessment) is a caries risk assessment and management system proposed by California Dental Association, which was then modified to the existing model; CAT is a caries-risk assessment and management protocols proposed by American Academy of Pediatric Dentistry; ADA (American Dental Association)

Table S3.Nyvad‘s criterion of caries diagnostic (1999)[23]

| Score Category | Criteria |
| --- | --- |
| 1. Sound | Normal enamel translucency and texture (slight staining allowed in otherwise sound fissure). |
| 1. Active caries   (intact surface) | Surface of enamel is whitish/yellowish opaque with loss of luster;  feels rough when the tip of the probe is moved gently across the surface; generally covered with plaque. No clinically detectable loss of substance.  Smooth surface: Caries lesion typically located close to gingival margin.  Fissure/pit: Intact fissure morphology; lesion extending along the walls of the fissure. |
| 1. Active caries   (surface discontinuity) | Same criteria as score 1. Localized surface defect (microcavity) in enamel only. No undermined enamel or softened floor detectable with the explorer. |
| 1. Active caries   (cavity) | Enamel/dentin cavity easily visible with the naked eye; surface of cavity feels soft or leathery on gentle probing. There may or may not be pulpal involvement. |
| 1. Inactive caries   (intact surface) | Surface of enamel is whitish, brownish or black. Enamel may be shiny and feels hard and smooth when the tip of the probe is moved gently across the surface. No clinically detectable loss of substance.  Smooth surface: Caries lesion typically located at some distance from gingival margin.  Fissure/pit: Intact fissure morphology; lesion extending along the walls of the fissure. |
| 1. Inactive caries   (surface discontinuity） | Same criteria as score 4. Localized surface defect (microcavity) in enamel only. No undermined enamel or softened floor detectable with the explorer. |
| 1. Inactive caries   (cavity) | Enamel/dentin cavity easily visible with the naked eye; surface of cavity may be shiny and feels hard on probing with gentle pressure. No pulpal involvement. |
| 1. Filling (sound surface) |  |
| 1. Filling + active caries | Caries lesion may be cavitated or non-cavitated. |
| 1. Filling + inactive caries | Caries lesion may be cavitated or non-cavitated. |

Table S4. ICDAS codes and criteria[6, 25]

| Code | Visual description | Code | Radiographic description |
| --- | --- | --- | --- |
| 0 | Sound tooth surface: No evidence of caries after 5 sec air drying | 0 | No radiolucency |
| 1 | First visual change in enamel: Opacity or discoloration (white or brown) is visible at the entrance to the pit or fissure seen after prolonged air drying | RA1 | Radiolucency in the outer half of the enamel |
| 2 | Distinct visual change in enamel visible when wet, lesion must be visible when dry | RA2 | Radiolucency in the inner half of the enamel including or not the DEJ (enamel-dentin junction) |
|  |  | RA3 | Radiolucency limited to the outer third of dentin |
| 3 | Localized enamel breakdown (without clinical visual signs of dentinal involvement) seen when wet and after prolonged drying | RA4 | Radiolucency reaching the middle third of dentin |
| 4 | Underlying dark shadow from dentine |  |  |
| 5 | Distinct cavity with visible dentine | RA5 | Radiolucency reaching the inner third of dentin |
| 6 | Extensive (more than half the surface) distinct cavity with visible dentine | RA6 | Radiolucency into the pulp |

Table S5. ICDAS-CAA criteria for carious lesion activity assessment[26, 27]

| Code | Description |
| --- | --- |
| ICDAS 1~3 | Active lesion: surface of enamel is whitish/yellowish opaque with loss of luster; feels rough when the tip of the probe is moved gently across the surface. Lesion is in a plaque stagnation area, i.e.: pits and fissures, near the gingival and approximal surface below the contact point. |
|  | Inactive lesion: surface of enamel is whitish, brownish or black. Enamel may be shiny and feels hard and smooth when the tip of the probe is moved gently across the surface. For smooth surfaces, the carious lesion is typically located at some distance from the gingival margin. |
| ICDAS 4 | Probably active. |
| ICDAS 5~6 | Active lesion: cavity feels soft or leathery on gently probing the dentin. |
|  | Inactive lesion: cavity may be shiny and feels hard on gently probing the dentin. |

Table S6. ADA Caries-risk Assessment Form（Age 0-6）

|  | **Low risk** | **Moderate risk** | **High risk** |
| --- | --- | --- | --- |
| **Contributing Conditions** | | | |
| 1. **Fluoride Exposure** (through drinking water, supplements, professional applications, toothpaste) | 口Yes | 口No |  |
| 2. **Sugary Foods or Drinks** (including juice, carbonated or non-carbonated soft drinks, energy drinks, medicinal syrups) | 口Primarily at mealtimes | 口Frequent or prolonged between meal exposures/day | 口Bottle or sippy cup with anything other than water at bed time |
| 3. **Eligible for Government Programs** (WIC, Head Start, Medicaid or SCHIP) | 口No |  | 口Yes |
| 4. **Caries Experience of Mother, Caregiver and/or other Siblings** | 口No carious lesions in last 24 months | 口Carious lesions in last 7-23 months | 口Carious lesions in last 6 months |
| 5. **Dental Home**: established patient of record in a dental office | 口Yes | 口No |  |
| **General Health Conditions** | | | |
| 1. **Special Health Care Needs** (developmental, physical, medical or mental disabilities that prevent or limit performance of adequate oral health care by themselves or caregivers) | 口No |  | 口Yes |
| **Clinical Conditions** | | | |
| 1. **Visual or Radiographically Evident Restorations/ Cavitated Carious Lesions** | 口No new carious lesions or restorations in last 24 months |  | 口Carious lesions or restorations in last 24 months |
| 2. **Non-cavitated** (incipient) **Carious Lesions** | 口No new lesions in last 24 months |  | 口New lesions in last 24 months |
| 3. **Teeth Missing Due to Caries** | 口No |  | 口Yes |
| 4. **Visible Plaque** | 口No | 口Yes |  |
| 5. **Dental/Orthodontic Appliances Present** (fixed or removable) | 口No | 口Yes |  |
| 6. **Salivary Flow** | 口Visually adequate |  | 口Visually inadequate |
| Overall assessment of dental caries risk: 口Low 口Moderate 口High | | | |

Low Risk = only conditions in “Low Risk” column present; Moderate Risk = only conditions in “Low” and/or “Moderate Risk” columns present; High Risk = one or more conditions in the “High Risk” column present.

Table S7. ADA Caries-risk Assessment Form（Age ＞6）

|  | **Low risk** | **Moderate risk** | **High risk** |
| --- | --- | --- | --- |
| **Contributing Conditions** | | | |
| 1. **Fluoride Exposure** (through drinking water, supplements, professional applications, toothpaste) | 口Yes | 口No |  |
| 2. **Sugary Foods or Drinks** (including juice, carbonated or non-carbonated soft drinks, energy drinks, medicinal syrups) | 口Primarily at mealtimes |  | 口Frequent or prolonged between meal exposures/day |
| 3. **Caries Experience of Mother, Caregiver and/or other Siblings** (for patients ages 6-14) | 口No carious lesions in last 24 months | 口Carious lesions in last 7-23 months | 口Carious lesions in last 6 months |
| 4. **Dental Home**: established patient of record, receiving regular dental care in a dental office | 口Yes | 口No |  |
| **General Health Conditions** | | | |
| 1. **Special Health Care Needs** (developmental, physical, medical or mental disabilities that prevent or limit performance of adequate oral health care by themselves or caregivers) | 口No | 口Yes (over age 14) | 口Yes (ages 6-14) |
| 2. **Chemo/Radiation Therapy** | 口No |  | 口Yes |
| 3. **Eating Disorders** | 口No | 口Yes |  |
| 4. **Medications that Reduce Salivary Flow** | 口No | 口Yes |  |
| 5. **V. Drug/Alcohol Abuse** | 口No | 口Yes |  |
| **Clinical Conditions** | | | |
| 1. **Cavitated or Non-Cavitated** (incipient)  **Carious Lesions or Restorations** (visually or  radiographically evident) | 口No new carious lesions or restorations in last 36 months | 口1-2 new carious lesions or restorations in last 36 months | 口3 or more new carious lesions or restorations in last 36 months |
| 2. **Teeth Missing Due to Caries in past 36 months** | 口No |  | 口Yes |
| 3. **Visible Plaque** | 口No | 口Yes |  |
| 4. **Unusual Tooth Morphology** that compromises  oral hygiene | 口No | 口Yes |  |
| 5. **Interproximal Restorations - 1 or more** | 口No | 口Yes |  |
| 6. **Exposed Root Surfaces** Present | 口No | 口Yes |  |
| 7. **Restorations with Overhangs** and/or **Open Margins; Open Contacts** with Food Impaction | 口No | 口Yes |  |
| 8. **Dental/Orthodontic Appliances** (fixed or removable) | 口No | 口Yes |  |
| 9．**Severe Dry Mouth (Xerostomia)** | 口No |  | 口Yes |
| Overall assessment of dental caries risk: 口Low 口Moderate 口High | | | |

Low Risk = only conditions in “Low Risk” column present; Moderate Risk = only conditions in “Low” and/or “Moderate Risk” columns present; High Risk = one or more conditions in the “High Risk” column present.

Table S8. AAPD Caries-risk Assessment Form for 0-5 Years Old

| **Factors** | **High risk** | **Moderate risk** | **Low risk** |
| --- | --- | --- | --- |
| **Risk factors, social/biological** | | | |
| 1.Mother/primary caregiver has active dental caries | ✓ |  |  |
| 2.Parent/caregiver has life-time of poverty, low health literacy | ✓ |  |  |
| 3.Child has frequent exposure (>3 times/day) between-meal sugar-containing snacks or beverages per day | ✓ |  |  |
| 4.Child uses bottle or non-spill cup containing natural or added sugar frequently, between meals and/or at bedtime | ✓ |  |  |
| 5.Child is a recent immigrant |  | ✓ |  |
| 6.Child has special health care needs |  | ✓ |  |
| **Protective factors** | | | |
| 1.Child receives optimally-fluoridated drinking water or fluoride supplements |  |  | ✓ |
| 2.Child has teeth brushed daily with fluoridated toothpaste |  |  | ✓ |
| 3.Child receives topical fluoride from health professional |  |  | ✓ |
| 4.Child has dental home/regular dental care |  |  | ✓ |
| **Clinical findings** | | | |
| 1.Child has non-cavitated (incipient/white spot) caries or enamel defects | ✓ |  |  |
| 2.Child has visible cavities or fillings or missing teeth due to caries | ✓ |  |  |
| 3.Child has visible plaque on teeth | ✓ |  |  |
| Circling those conditions that apply to a specific patient helps the practitioner and parent understand the factors that contribute to or protect from caries. Risk assessment categorization of low, moderate, or high is based on preponderance of factors for the individual. However, clinical judgment may justify the use of one factor (e.g., frequent exposure to sugar-containing snacks or beverages, more than one decayed missing filled surfaces [dmfs]) in determining overall risk. | | | |
| Overall assessment of the child’s dental caries risk: High口 Moderate口 Low口 | | | |

Table S9. AAPD Caries-risk Assessment Form for ≥6 Years Old(For Dental Providers)

| **Factors** | **High risk** | **Moderate risk** | **Low risk** |
| --- | --- | --- | --- |
| **Risk factors, social/biological** | | | |
| 1.Patient has life-time of poverty, low health literacy | ✓ |  |  |
| 2.Patient has frequent exposure (>3 times/day) between-meal sugar-containing snacks or beverages per day | ✓ |  |  |
| 3.Child is a recent immigrant |  | ✓ |  |
| 4.Patient has special health care needs |  | ✓ |  |
| **Protective factors** | | | |
| 1.Patient receives optimally-fluoridated drinking water |  |  | ✓ |
| 2.Patient brushes teeth daily with fluoridated toothpaste |  |  | ✓ |
| 3.Patient receives topical fluoride from health professional |  |  | ✓ |
| 4.Patient has dental home/regular dental care |  |  | ✓ |
| **Clinical findings** | | | |
| 1.Patient has ≥1 interproximal caries lesions | ✓ |  |  |
| 2.Patient has active non-cavitated (white spot) caries lesions or enamel defects | ✓ |  |  |
| 3.Patient has low salivary flow | ✓ |  |  |
| 4.Patient has defective restorations |  | ✓ |  |
| 5.Patient wears an intraoral appliance |  | ✓ |  |
| Circling those conditions that apply to a specific patient helps the practitioner and patient/parent understand the factors that contribute to or protect from caries. Risk assessment categorization of low, moderate, or high is based on preponderance of factors for the individual. However, clinical judgment may justify the use of one factor (e.g., interproximal lesions, low salivary flow) in determining overall risk. | | | |
| Overall assessment of the dental caries risk: High口 Moderate口 Low口 | | | |

Table S10. Caries Risk Assessment Form — Children Age 6 and Over/Adults

| **Disease Indicators (Any one “YES” signifies likely “High Risk” and to do a bacteria test**)** | **High risk** | **Moderate risk** | **Low risk** |
| --- | --- | --- | --- |
| 1.Visible cavities or radiographic penetration of the dentin | Yes |  |  |
| 2.Radiographic approximal enamel lesions (not in dentin) | Yes |  |  |
| 3.White spots on smooth surfaces | Yes |  |  |
| 4.Restorations last 3 years | Yes |  |  |
| **Risk Factors (Biological predisposing factors)** | | | |
| 1.MS and LB both medium or high (by culture**) |  | Yes |  |
| 2.Visible heavy plaque on teeth |  | Yes |  |
| Frequent snack (> 3x daily between meals) |  | Yes |  |
| 3.Deep pits and fissures |  | Yes |  |
| 4.Recreational drug use |  | Yes |  |
| 5.Inadequate saliva flow by observation or measurement (**If measured, note the flow rate below) |  | Yes |  |
| 6.Saliva reducing factors (medications/radiation/systemic) |  | Yes |  |
| 7.Exposed roots |  | Yes |  |
| 8.Orthodontic appliances |  | Yes |  |
| **Protective Factors** | | | |
| 1.Lives/work/school fluoridated community |  |  | Yes |
| 2.Fluoride toothpaste at least once daily |  |  | Yes |
| 3.Fluoride toothpaste at least 2x daily |  |  | Yes |
| 4.Fluoride mouthrinse (0.05% NaF) daily |  |  | Yes |
| 5.5,000 ppm F fluoride toothpaste daily |  |  | Yes |
| 6.Fluoride varnish in last 6 months |  |  | Yes |
| 7.Office F topical in last 6 months |  |  | Yes |
| 8.Chlorhexidine prescribed/used one week each of last 6 months |  |  | Yes |
| 9.Xylitol gum/lozenges 4x daily last 6 months |  |  | Yes |
| 10.Calcium and phosphate paste during last 6 months |  |  | Yes |
| 11.Adequate saliva flow (> 1 ml/min stimulated) |  |  | Yes |
| (EXTREME RISK = HIGH RISK + SEVERE SALIVARY GLAND HYPOFUNCTION)  CARIES RISK ASSESSMENT (CIRCLE): EXTREME HIGH MODERATE LOW | | | |
